# Interdisciplinary 3D digital treatment simulation before complex esthetic rehabilitation of orthodontic, orthognathic and prosthetic treatment: workflow establishment and primary evaluation

**DOI:** 10.1186/s12903-022-02070-z

**Published:** 2022-02-11

**Authors:** Longwei Lv, Wei He, Hongqiang Ye, Kwantong Cheung, Lin Tang, Shimin Wang, Lang You, Chunlei Xun, Yongsheng Zhou

**Affiliations:** 1 Department of Prosthodontics, Peking University School and Hospital of Stomatology & National Center of Stomatology & National Clinical Research Center for Oral Disease & National Engineering Research Center of Oral Biomaterials and Digital Medical Devices & Beijing Key Laboratory of Digital Stomatology, Key Laboratory of Digital Stomatology & Research Center of Engineering and Technology for Computerized Dentistry Ministry of Health & NMPA Key Laboratory for Dental Materials, Beijing, People’s Republic of China; 2 Department of Oral Maxillofacial Surgery, Peking University School and Hospital of Stomatology, 22 Zhongguancun Avenue South, Haidian District, Beijing, 100081 People’s Republic of China; 3 Department of Orthodontics, Peking University School and Hospital of Stomatology, 22 Zhongguancun Avenue South, Haidian District, Beijing, 100081 People’s Republic of China

**Keywords:** Interdisciplinary treatment, Digitalization, Simulated treatment plan, Orthodontics and orthognathics, Prosthodontics, Esthetic rehabilitation

## Abstract

**Background:**

An interdisciplinary treatment simulation and smile design before a complex esthetic rehabilitation is important for clinicians’ decision-making and patient motivation. Meanwhile, intervention and interaction are necessary for dental specialists in these complex rehabilitations. However, it is difficult to visualize an interdisciplinary treatment plan by using the conventional method, especially when orthognathic surgery is involved, thus hindering communication between dental specialists. This research aims to establish a 3D digital workflow of interdisciplinary treatment simulation to solve this problem.

**Methods:**

An interdisciplinary 3D digital workflow of simulated treatment plan for complex esthetic rehabilitation was established. Eleven patients were enrolled and illustrated with their treatment plans using 3D treatment simulation, as well as 2D digital smile design (DSD) plus wax-up. Visual analogue scales (VAS) were used to rate the intuitiveness, understanding, and satisfaction or help between the two methods by patients and dental specialists.

**Results:**

According to the ratings from the patients, 3D treatment simulation showed obvious advantages in the aspects of intuitiveness (9.7 ± 0.5 vs 6.4 ± 1.4) and treatment understanding (9.1 ± 0.8 vs 6.6 ± 1.5), and the satisfaction rates were also higher (9.0 ± 0.6 vs 7.1 ± 1.8). Dental specialists regarded the 3D digital plans as more intuitive (8.9 ± 0.8 vs 5.9 ± 1.0) and useful to understand the plans from the other specialists (8.9 ± 0.7 vs 6.1 ± 1.0) and helpful to their own treatment plans (8.7 ± 0.9 vs 5.9 ± 1.4).

**Conclusions:**

The interdisciplinary 3D digital treatment simulation helps both patients and dental specialists to improve treatment understanding, and facilitates dental specialists for decision-making before complex esthetic rehabilitation.

***Trial registration*:**

This study was registered in the National Clinical Trials Registry under the identification number MR-11-20-002862. This is an observational study in which we did not assign the intervention.

**Supplementary Information:**

The online version contains supplementary material available at 10.1186/s12903-022-02070-z.

## Background

Complex oral-maxillofacial esthetic defects, which can be caused by various reasons such as genetic factors, growth and development factors, trauma, often lead to multiple problems ranging from tooth and dentition defects, malocclusion, to maxillofacial deformities [1–4]. As for these complex cases, the most challenging phase of their treatment is comprehensive diagnosis, planning and decision-making by the interdisciplinary team before the whole treatment starts [5, 6]. However, it is difficult to obtain a visible and overall interdisciplinary treatment plan, including prosthodontics, orthodontics and orthognathic surgery at the same time, through conventional methods based on photographs, model casts and wax-up [5].

In order to provide a visible treatment simulation and predictive effect before the interdisciplinary treatment starts, 2-dimensional (2D) digital smile design (DSD), the existing gold standard for esthetic smile design through prosthetic simulation, has been introduced to the process of interdisciplinary treatment planning [7, 8]. DSD is an effective method in prosthodontic and periodontic interdisciplinary treatment, and can also help orthodontic treatment planning to some extent [7, 8]. However, it is impossible to use the DSD method to accomplish an orthognathic simulation [6, 9]. Meanwhile, DSD can only guide the orthodontic plan of the upper anterior teeth, unable to provide a treatment simulation of the complete dentition [7]. In addition, this 2D method is inevitably influenced by the angulation of photograph taking, thus affecting its accuracy [10].

The emerging technology of 3-dimensional (3D) digitalization might be a possible solution for the overall treatment simulation before the whole complex interdisciplinary treatment starts. However, there have been few reports on the overall 3D interdisciplinary treatment planning up till now [9]. Several cases used 3D dental software to benefit the interdisciplinary treatment during the process of treatment execution [11–13], but there have been few workflows to simulate the whole interdisciplinary treatment process for patient education and clinician communication before the treatment starts, particularly when orthognathic surgery is needed in the interdisciplinary treatment process. In addition, there has been no research to evaluate the efficacy of the 3D interdisciplinary workflow [9]. Moreover, the incompatibility among distinct 3D dental software developed by different companies may also hinted their usage by different dental specialists in the interdisciplinary team. Therefore, a visible and measurable interdisciplinary 3D workflow that bridges different dental specialties for comprehensive treatment planning has been urgently expected, which will not only serve as a useful platform for communication and decision-making among dental specialists in the interdisciplinary team, but also provide an effective communication tool between clinicians and patients.

We herein proposed an interdisciplinary 3D fully-digital workflow for the treatment planning of complex esthetic rehabilitation by combining professional 3D dental software and a general 3D software. A general 3D software, Geomagic Studio, was utilized as a universal platform to establish 3D virtual patient, to combine treatment plans from different dental specialists, and to view the treatment process and predicted effect, thus solving the problem of incompatibility among different dental software. Three dental software (3Shape Dental System, 3Shape Ortho Analyzer, and Proplan) was used for prosthodontic, orthodontic, and orthognathic plans. These three kinds of software are all treatment planning and execution software, so that the treatment plans can be easily moved on to the treatment execution process without complex transfer. In addition, the efficacy of the proposed interdisciplinary 3D workflow was validated in a series of complex interdisciplinary cases, and was evaluated by both patients and dental specialists.

## Methods

### Recruitment and eligibility criteria

The study was carried out in the Departments of Prosthodontics, Orthodontics, and Oral Maxillofacial Surgery in Peking University School and Hospital of Stomatology hospital. The inclusion criteria were as follows: (1) Patients who were at least 18 years old, and had good general health; (2) Patients with complex esthetic problems, including malocclusion, dento-maxillofacial deformities, as well as tooth defect or deformed tooth or tooth loss in the upper or upper and lower anterior segment, who needed interdisciplinary treatment from at least three specialties, including orthodontics, orthognathic surgery, and prosthodontics; (3) Patients who intend to solve their esthetic problems by orthodontic, orthognathic, and prosthetic treatment. In order to control the variation and guarantee the comparability among different cases, patients whose treatment plans would be orthognathic surgery first before orthodontic treatment were excluded. Subjects with any cognitive incapacity or medical disorders were excluded. The sample size (n = 11) was calculated based on the preliminary results with an α of 5% and a power of 90%. Eleven patients (2 males and 9 females, aged from 19 to 36 years old) who came to any of the above three departments from October 2019 to October 2020, were recruited in this research.

### Study design, settings, and experimental procedure

The self-controlled experiment was applied. There were two methods to illustrate the treatment process and predicted effect to each patient: Method (1) was 3D digital simulated treatment plan; Method (2) was DSD plus wax-up. Random numbers were generated by the random number table and were sealed in envelops and the envelops were opened in the order of enrolment. The order of illustration, Method (1)-Method (2), or Method (2) -Method (1), was decided by the number in the envelop. An odd number means illustrating the Method (1) 3D digital simulated treatment plan first. Subsequently, each patient was asked to fill in a questionnaire to evaluate the intuitiveness of the treatment plan, their understanding of the treatment process, and their satisfaction to the treatment plan. Nine dental specialists, 3 specialists from each specialty, who are all senior dental specialists, were also asked to fill in a questionnaire to evaluate the intuitiveness of the treatment plans, their understanding of the treatment plans from the other specialists in the team, and the help of the two methods to their own treatment plans. Consent forms were signed before the study.

A visual analogue scale (VAS) was used to quantify the subjective evaluations. The VAS scores ranged from 0 to 10, with the leftmost point indicating “very poor” and the rightmost “very good”. The distance between the origin point and the mark point scored by patient or dental specialist was measured by the same operator, and the data was converted into an evaluation score based on the established standard of VAS [14, 15].

### 2D digital smile design (DSD) and wax-up

**Fig. 1:**
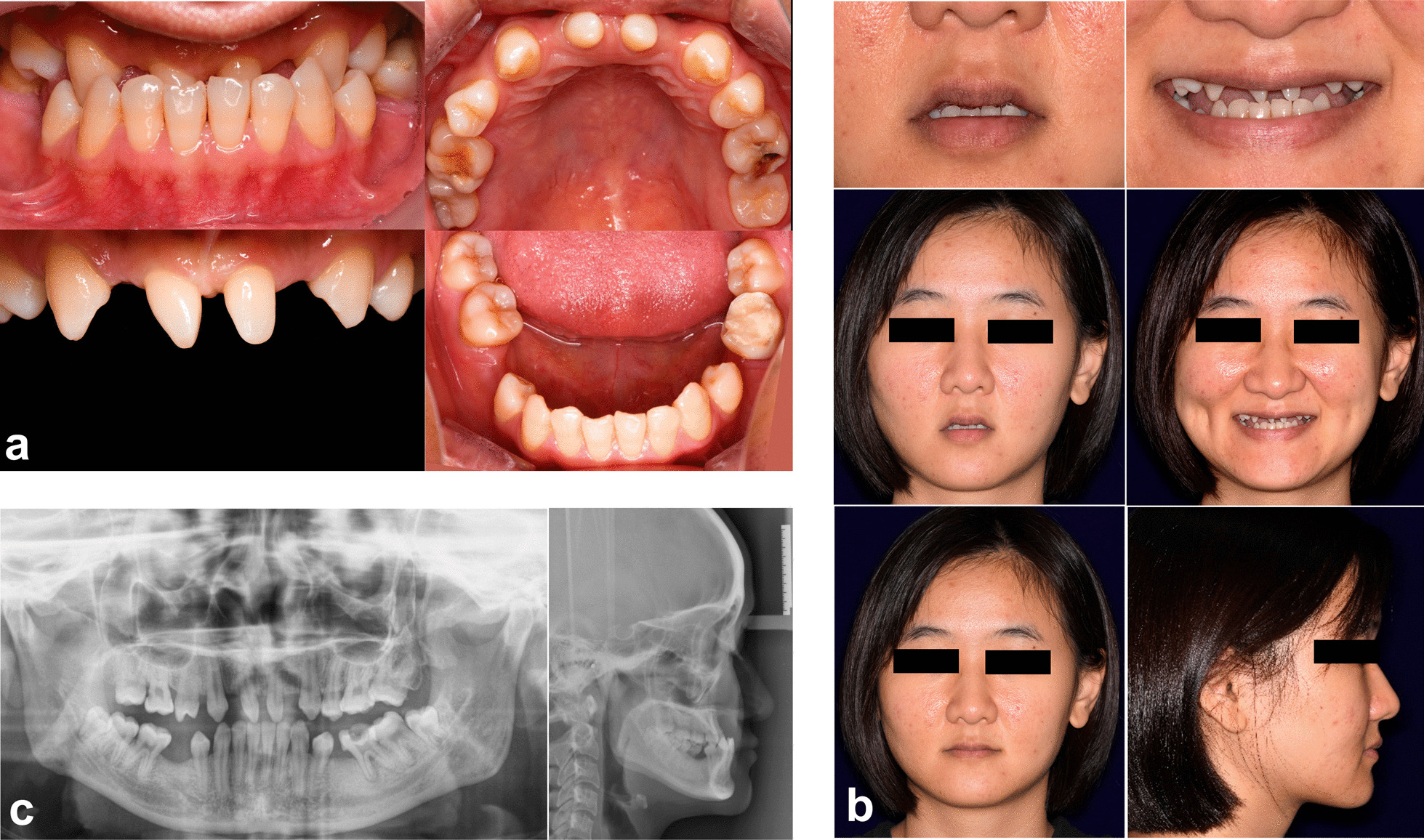
Initial intraoral, facial photographs and radiological examination. **a** Intraoral photographs; **b** facial photographs in rest position, wide smile, closed lips, and profile; c. panoramic radiography and cephalogram

*Step 1. Photography and impression taking* The exemplary case showed a patient with congenital missing teeth, deformed teeth, maxillary retrognathism and mandibular prognathism (Fig. 1). Intraoral (Fig. 1a) and facial photographs (Fig. 1b) of the patient were made according to the requirement of DSD. Radiological examinations, including panoramic radiography and cephalogram, were made (Fig. 1c). Maxillary and mandible impressions were taken by silicone rubber and plaster model casts were made.

**Fig. 2:**
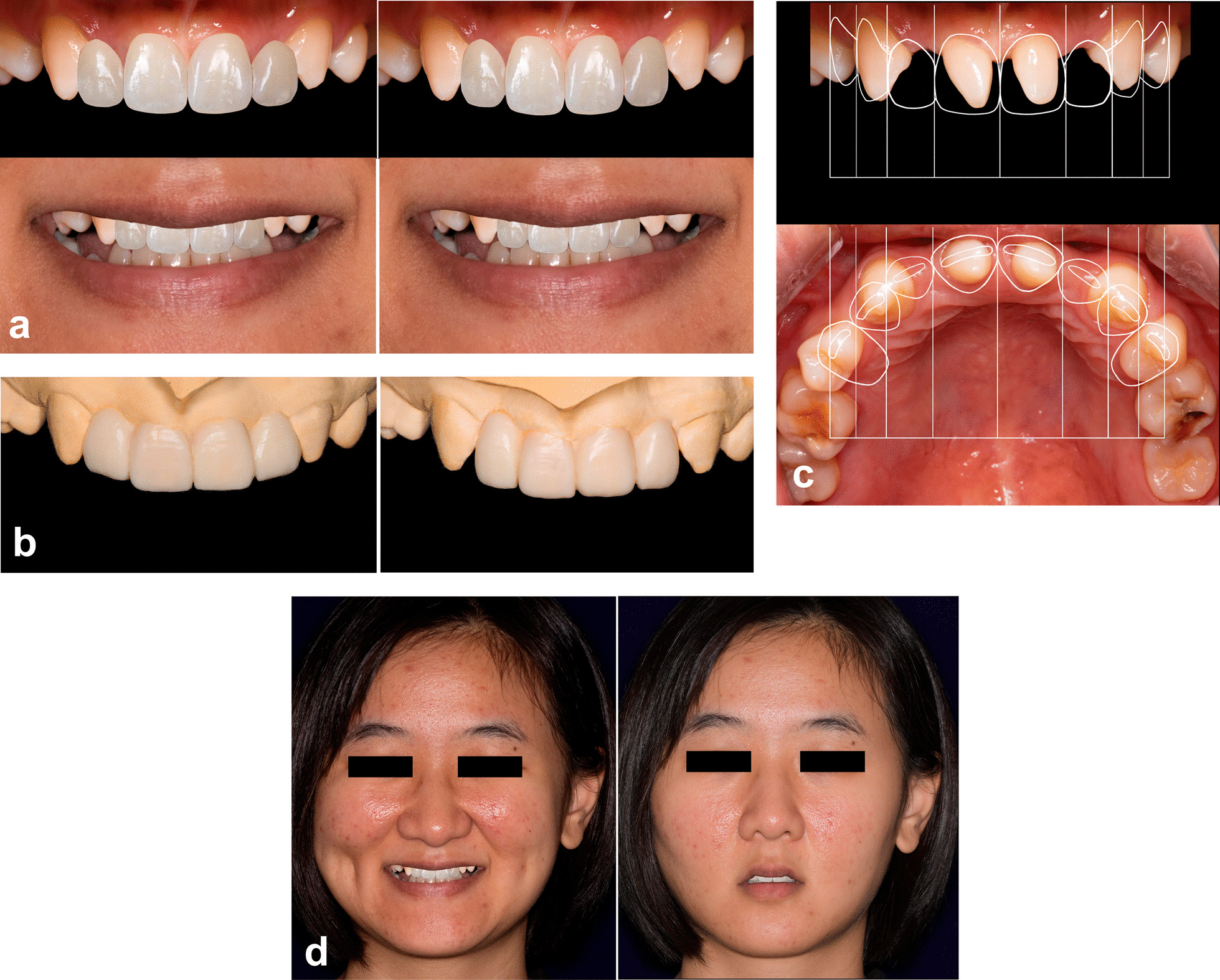
Treatment plan of 2D digital smile design (DSD) and conventional wax-up. **a** Prosthodontic designs with different wide-length ratio of the anterior teeth on photographs; **b** wax-ups of the prosthodontic designs (this patient preferred the design on the left); **c** orthodontic design of the anterior teeth according to the prosthodontic design the patient chose; **d** predicted effect of wide smile and rest position on facial photographs

*Step 2. Case analyses, DSD and wax-up* The interdisciplinary team performed dento-facial analyses and discussed a primary treatment design. Then the prosthodontic specialist designed several sets of prostheses with different wide-length ratio of the anterior teeth on photographs (Fig. 2a) according to established protocol of DSD [16]. The incisal edge was designed referring to both the width-length ratio of the incisors and the experience from orthognathic specialist how the upper lip would change after surgery. Wax-ups were made in accordance with DSD (Fig. 2b). Patient participated in the selection of the form and wide-length ratio of their prostheses, and chose the prosthodontic plan. Then, the orthodontic specialist performed the orthodontic DSD on photographs according to the prosthodontic plan the patient chose (Fig. 2c).

*Step 3. Illustration to the patient* DSD photographs of prosthodontic design (Fig. 2a), the wax-ups (Fig. 2b), orthodontic design (Fig. 2c) and predicted facial graphs (Fig. 2d) were shown to the patients to illustrate the treatment procedure and predictive effect.

### Interdisciplinary 3D digital workflow protocol

**Fig. 3:**
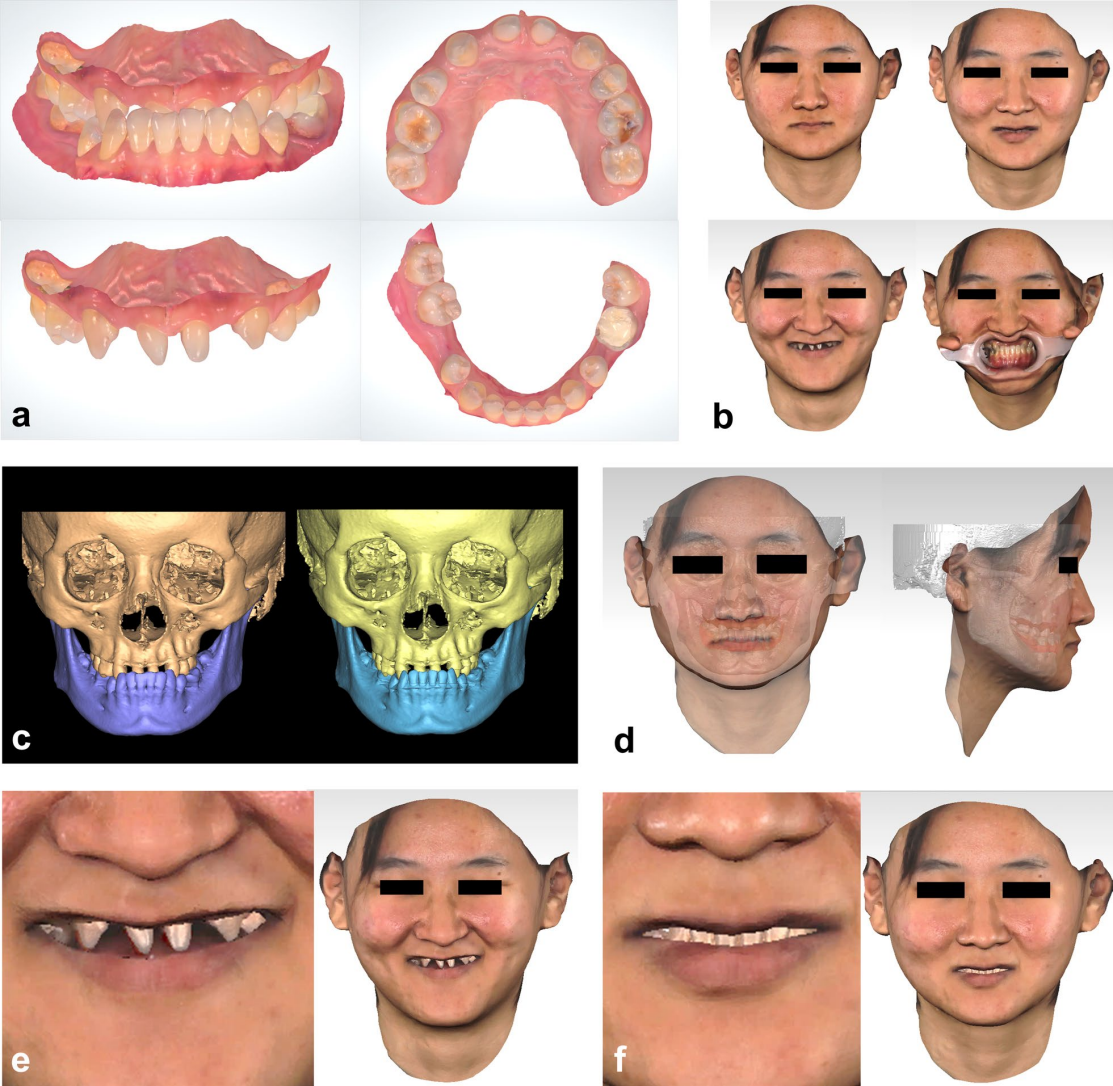
Initial 3D data acquisition and patient digitalization. **a** 3D dentitions; **b** 3D facial photographs in rest position, wide smile, closed lips and maximum intercuspal position; **c** 3D information of oral-maxillofacial bones from CBCT (left) and the teeth are replaced with 3D dentitions (right); **d**–**f** 3D virtual patient in closed lips (**d**), wide smile (**e**) and rest position (**f**)

*Step 1. Data acquisition and patient digitalization* The intraoral 3D information was obtained by an intraoral scanner (TRIOS; 3Shape, Copenhagen, Denmark) and was transformed from DCM files with color into VRML files with color in 3Shape Ortho Analyzer (3Shape) (Fig. 3a). 3D dynamic facial information in closed lips, rest position, wide smile, and the maximum intercuspal position was obtained by Face Scan (3D-SHAPE, Denmark) in OBJ files with color (Fig. 3b). Cone beam computed tomography (CBCT, NewTom VG; Quantitative Radiology, Verona, Italy) was scanned with a scan range from the supraorbital margin to the inferior margin of the mandible and a head position to ensure the Frankfort plane and the interpupillary line parallel to the horizontal plane, and 3D information of oral-maxillofacial bones were obtained by Mimics 22.0 (Materialise, Belgium) in STL files (Fig. 3c left). Subsequently, the 3D dentition, the 3D facial images, the 3D maxilla and mandible, and were imported into Geomagic Studio. To establish a 3D virtual patient with color information (Fig. 3d-f), the 3D dentition and facial images were registered to the 3D bones, and the tooth information in facial images and bones were substituted by 3D dentitions (Fig. 3c right, Fig. 3e, f). All these files were stored in a cloud platform and shared with the patient’s own interdisciplinary team.

*Step 2. Case analyses and comprehensive 3D planning* The interdisciplinary team performed dento-facial analyses, shared their digital brainstorm, discussed a primary treatment design, and decided the sequence of the 3D treatment plan, which could be either coincident or different with the actual treatment sequence. Then, according to the agreed sequence, the first specialist performed a digital treatment simulation and uploaded the 3D treatment plan to the cloud platform, and the next specialist downloaded the data and performed subsequent treatment plan, and so on. Revisions could be made by the previous specialists when other specialists raised the requirements during their treatment plan. Prosthodontic, orthodontic, and orthognathic treatment plans were designed using 3Shape Dental System (3Shape), 3Shape Ortho Analyzer (3Shape), and Proplan CMF 3.0 (Materialise) respectively, while Geomagic Studio was used to import the design in the 3D virtual patient.

**Fig. 4:**
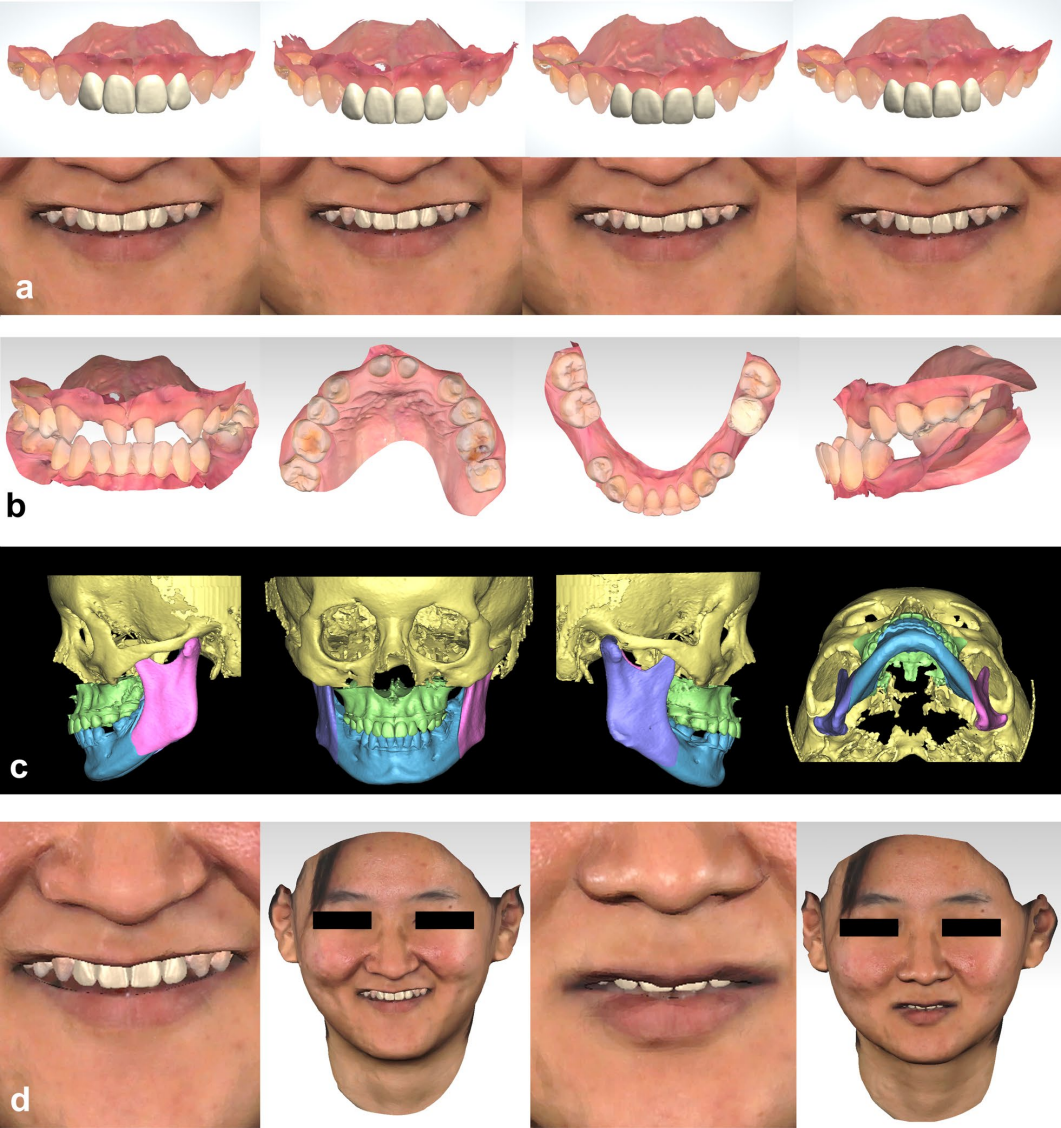
Interdisciplinary 3D digital treatment simulation. **a** 3D prosthodontic designs with different wide-length ratio of the anterior teeth (up) and predicted effect in wide smile (down); **b** orthodontic simulation according to the prosthodontic design the patient chose; **c** simulated orthognathic surgery; **d** predicted effect of wide smile and rest position

For example, the actual treatment procedure of the exemplary case would be orthodontic treatment, orthognathic surgery, and prosthesis. After case analyses and team discussion, the orthognathic specialist designed the preliminary orthognathic surgical plan first, recorded the movements and rotations of the maxilla and mandible. Under the instruction of the incisal exposure after preliminary orthognathic plan, prosthodontic specialist designed several sets of prostheses with different wide-length ratio of the anterior teeth (Fig. 4a). Patient participated in the selection of the form and wide-length ratio of their prostheses, and chose the prosthodontic plan (the second from left in Fig. 4a). Subsequently, the orthodontic specialist performed the orthodontic treatment simulation (Fig. 4b) according to the protheses the patient chose, then the orthognathic specialist made minor refinements on the preliminary orthognathic plan, and lastly the prosthodontic specialist imported the protheses in the dentition after orthognathic simulation and made minor refinements (Fig. 4c).

*Step 3. Illustration to the patient* The interdisciplinary 3D treatment simulation was illustrated to the patient in line with the actual treatment process in Geomagic Studio by using the 3D virtual patient. The patient could see both the interdisciplinary treatment procedure (Fig. 4a–c, Additional file 1) and the 3D digital smile design (Fig. 4d).

### Statistical analysis

SPSS software (IBM, SPSS, Statistics 25.0) was used for statistical analysis. Paired t-test was performed and the level of significance was set at *P* < 0.05. Descriptive statistics were presented as means and standard deviations.

## Results

According to the ratings from the patients, 3D digital simulated treatment plan showed obvious advantages in the aspects of intuitiveness and understanding of the treatment plan, and the satisfaction rates were also higher than DSD plus conventional wax-up. Specifically, as shown in Table 1, the average VAS score on the intuitiveness of the 3D treatment plans (9.7 ± 0.5) was significantly higher than DSD plus conventional wax-up (6.4 ± 1.4) (*P* < 0.01); the average VAS score on the understanding of the 3D treatment plan (9.1 ± 0.8) was significantly higher than DSD plus conventional wax-up (6.6 ± 1.5) (*P* < 0.01); and the satisfaction rates of the patients to the 3D treatment plan (9.0 ± 0.6) were also significantly higher than DSD plus conventional wax-up (7.1 ± 1.8) (*P* < 0.01).

**Table 1:** Mean values and standard deviations (SD) of the VAS scores of intuitiveness, understanding and satisfaction of the treatment plans rated by the patients (*P < 0.05, **P < 0.01)

	3D digital treatment plan	DSD plus conventional wax-up	*P* value
Intuitiveness	9.7 ± 0.5**	6.4 ± 1.4	< 0.001
Understanding	9.1 ± 0.8**	6.6 ± 1.5	< 0.001
Satisfaction	9.0 ± 0.6**	7.1 ± 1.8	0.004

As for ratings from dental specialists, 3D digital simulated treatment plan was also superior in the aspects of intuitiveness and understanding of the treatment plan. More importantly, the 3D treatment plan may better help dental specialists for their own design and treatment. Specifically, as shown in Table 2, the average VAS score on the intuitiveness of the 3D treatment plans (8.9 ± 0.8) was significantly higher than DSD plus conventional wax-up (5.9 ± 1.0) (*P* < 0.01); the average VAS score on the understanding of the 3D treatment plans (8.9 ± 0.7) was significantly higher than DSD plus conventional wax-up (6.1 ± 1.0) (*P* < 0.01); and the help which the dental specialists acquired from the 3D treatment plans (8.7 ± 0.9) was also more significant than DSD plus conventional wax-up (5.9 ± 1.4) (*P* < 0.01).

**Table 2:** Mean values and standard deviations (SD) of the VAS scores of intuitiveness, understanding and help of the treatment plans rated by dental specialists (*P < 0.05, **P < 0.01)

	3D digital treatment plan	DSD plus conventional wax-up	*P* value
Intuitiveness	8.9 ± 0.8**	5.9 ± 1.0	< 0.001
Understanding	8.9 ± 0.7**	6.1 ± 1.0	< 0.001
Help	8.7 ± 0.9**	5.9 ± 1.4	< 0.001

While analysing the results from the aspects of prosthodontic, orthodontic, and orthognathic specialists respectively (Table 3), we found that the average score on the intuitiveness of 3D treatment plan from orthognathic specialists (8.5 ± 1.0) was lower than prosthodontic (9.2 ± 0.5), and orthodontic specialists (9.0 ± 0.6). All specialists from the three specialties regarded the interdisciplinary 3D treatment plan help them to reach more thorough understanding of the treatment plan from the other specialists compared to DSD plus wax-up, and helped their decision-making in their own specialty.

**Table 3:** Mean values and standard deviations (SD) of the VAS scores of intuitiveness, understanding and help of the treatment plans rated by dental specialists and analysed with respect to prosthodontic, orthodontic and orthognathic specialists respectively. (*P < 0.05, **P < 0.01)

	3D digital treatment plan	DSD plus conventional wax-up	*P* value
*Prosthodontic specialists*			
Intuitiveness	9.2 ± 0.5**	6.0 ± 1.4	< 0.001
Understanding	9.1 ± 0.5**	6.1 ± 1.1	< 0.001
Help	8.7 ± 1.0**	5.6 ± 1.5	< 0.001
*Orthodontic specialists*			
Intuitiveness	9.0 ± 0.6**	6.0 ± 0.8	< 0.001
Understanding	8.9 ± 0.7**	6.2 ± 1.0	< 0.001
Help	8.8 ± 0.7**	6.2 ± 1.2	< 0.001
*Orthognathic specialists*			
Intuitiveness	8.5 ± 1.0**	5.8 ± 0.8	< 0.001
Understanding	8.8 ± 0.8**	6.0 ± 0.9	< 0.001
Help	8.7 ± 1.0**	5.9 ± 1.4	< 0.001

## Discussion

In this study, a 3D digital workflow for interdisciplinary treatment simulation was successfully established, which will not only serve as a useful platform for communication and decision-making among dental specialists in the interdisciplinary team, but also provide an effective communication tool between clinicians and patients. It allows to motivate the patients for treatment adhesion and to discuss their treatment process. Meanwhile, the patients were clarified that the simulation of the predicted results were not always identical to the final results of the treatment.

Even though 3D digital treatment simulation has been well-explored in each dental specialty respectively [17–21], reports on interdisciplinary 3D digital workflow have still been limited due to the incompatibility of distinct software from different companies. Recently, several interdisciplinary cases that involve two disciplines have been reported. For example, Punj et al. reported an interdisciplinary case of non-syndromic tooth agenesis, while Gonzaga et al. reported a case of ectodermal dysplasia, utilizing digital tools that involved in orthodontics and oral implantation [1,11]. Poggio et al. reported a full mouth rehabilitation using digital tools of prosthodontic and oral implantation [22]. Li et al. and Liu et al. have established and evaluated the interdisciplinary 3D digital workflow of two disciplines, including prosthodontics and periodontics [23], as well as prosthodontics and orthodontics [24]. Lately, Coachman et al. have classified the various existing digital software and illustrated their potential usage in the interdisciplinary cases [5]. This software classification has provided an important guideline for software selection in different stages of interdisciplinary treatment. However, the smile design for interdisciplinary treatment of orthognathic patient was still not mentioned. Herein, we established a 3D digital workflow for interdisciplinary treatment simulation and 3D smile design for complex esthetic rehabilitation of at least three dental specialties, including orthognathic surgery. To solve the problem of software incompatibility, the general 3D software Geomagic Studio was utilized as a universal platform to establish 3D virtual patient, to communicate, and to illustrate. The professional dental 3D software with the function of both treatment planning and execution software were chosen to ease the progress from treatment planning to treatment execution process without transfer. And more importantly, the efficacy of this 3D digital workflow was evaluated.

This study confirms the necessity and advantages of the proposed interdisciplinary 3D digital workflow in the following aspects. Firstly, it provides a visible treatment process and predictive effect before the whole interdisciplinary treatment starts which cannot be realized by DSD or wax-up. It is indeed more intuitive and easier to understand according to the ratings from both patients and dental specialists. Secondly, patients are generally more satisfied with their 3D digital simulated treatment plan, and better comprehension and satisfaction rate will significantly increase the compliance of the patient which is especially important in this long and complex treatment process. Thirdly, this interdisciplinary 3D digital workflow can better help dental specialists to understand the needs and plans of their co-workers and help their own decision-making, thus increasing the working efficiency of the interdisciplinary team.

In this study, interdisciplinary 3D digital workflow was evaluated by comparing with DSD plus conventional wax-up. DSD and conventional wax-up together was taken as control because of the following three reasons. Firstly, DSD has been regarded as the existing gold standard of esthetic digital smile design [6]. Secondly, in spite of prosthodontic design, DSD was recently reported useful to aid the orthodontic and interdisciplinary treatment plan [7, 8]. Thirdly, DSD is a 2D method, thus conventional wax-up and model cast was used together to increase the intuitiveness of the treatment simulation. Therefore, we made the most use of the conventional methods for treatment plan as control, avoiding exaggerating the effect of 3D digital workflow.

However, there are still some points to be improved in this workflow mainly due to the limitation of the existing hardware and software. Firstly, patients who were not satisfied enough with their 3D treatment plan complained of their unnatural facial expression and a lack of hair information in the face scan. Secondly, orthognathic specialist considered the interdisciplinary 3D treatment plan less intuitive compared with prosthodontic, orthodontic specialists because soft tissue prediction with colored facial information after orthognathic surgery cannot be realized by existing software based on CBCT data [25, 26]. Thirdly, compared with conventional method of DSD and wax-up, this digital workflow is still time-consuming and experience-dependent.

To summarize, this interdisciplinary 3D digital workflow can help dental specialists better understand the needs and plans of their co-workers and are able to make optimal plans before the treatment begins. On the other hand, this interdisciplinary workflow will also help the patient to better comprehend the treatment process, and benefit from improved treatment efficiency and better treatment effects from the interdisciplinary teams. However, the hardware of face scan is expected to be improved in the aspects of higher resolution and less exposure time. The software of colored and more precise soft tissue prediction after orthognathic surgery are expected. A universal platform which can realize treatment simulation and execution of all dental specialties is urgently expected to improve the efficiency of interdisciplinary treatment.

## Conclusions

An interdisciplinary 3D Digital treatment simulation workflow for complex esthetic rehabilitation of orthognathic patient was successfully established. This interdisciplinary 3D digital treatment simulation helps both patients and dental specialists to improve treatment understanding, and facilitates dental specialists for decision-making before complex esthetic rehabilitation.

## Supplementary Information

**Additional file 1**. The interdisciplinary 3-Dimensional treatment simulation.

## Data Availability

All data generated or analysed during this study are included in this published article.

## Abbreviations

2D: 2-Dimensional
3D: 3-Dimensional
DSD: Digital smile design
VAS: Visual analogue scales
